# Supplementary material for: MetaRibo-Seq measures translation in microbiomes
Source: Nat Commun. 2020 Jun 29;11:3268. doi: 10.1038/s41467-020-17081-z (PMC7324362; doi:10.1038/s41467-020-17081-z)
Supplement: Supplementary file 10 — Supplementary Data 7 [file 41467_2020_17081_MOESM10_ESM.zip › File2/Confidence_VeryHigh_Taxonomy/89935_out.krona.html]

Javascript must be enabled to view this page.

members
magnitude
magnitudeUnassigned
count
unassigned
taxon
rank

89935\_out

14

1

SRS011084\_contig\_number\_contig-100\_13652.120221

2
13
superkingdom

phylum
976
1

200643
1
class

1
171549

SRS055017\_contig\_number\_4200
order

phylum
204428
1

1
204429
class

order
1
51291

family
1
809

1
810
genus

species
813
1

SRS142599\_contig\_number\_contig-100\_3151.96935

1224
2
phylum

class
28211
2

2
204457
order

family
2
41297

genus
2
150203

2
2217673

SRS014235\_contig\_number\_48983SRS019685\_contig\_number\_38447
species

phylum
9
1239

9
186801
class

order
186802
2

SRS144506\_contig\_number\_57460SRS144537\_contig\_number\_39276
9

541000
1
family

1
216851
genus

1946510
1

SRS148196\_contig\_number\_27162
species

family
186803
2

species

SRS018888\_contig\_number\_3435SRS1041138\_contig\_number\_9915
1952134
2


SRS055017\_contig\_number\_contig-100\_243.205049
1
1897049
species

family
3
31979

genus
3
580596

species

SRS104636\_contig\_number\_2499SRS147139\_contig\_number\_contig-100\_4866.37436SRS147271\_contig\_number\_42409
3
2292298
